# TRAIP is involved in chromosome alignment and SAC regulation in mouse oocyte meiosis

**DOI:** 10.1038/srep29735

**Published:** 2016-07-11

**Authors:** Yi-Feng Yuan, Yi-Xin Ren, Peng Yuan, Li-Ying Yan, Jie Qiao

**Affiliations:** 1 Center for Reproductive Medicine, Department of Obstetrics and Gynecology, Peking University Third Hospital, No. 49 North HuaYuan Road, HaiDian District, Beijing 100191, China; 2 Key Laboratory of Assisted Reproduction, Ministry of Education, Beijing 100191, China

## Abstract

Recent whole-exome sequencing (WES) studies demonstrated that TRAIP is associated with primordial dwarfism. Although TRAIP was partially studied in mitosis, its function in oocyte meiosis remained unknown. In this study, we investigated the roles of TRAIP during mouse oocyte meiosis. TRAIP was stably expressed during oocytes meiosis and co-localized with CREST at the centromere region. Knockdown of TRAIP led to DNA damage, as revealed by the appearance of γH2AX. Although oocytes meiotic maturation was not affected, the proportions of misaligned chromosomes and aneuploidy were elevated after TRAIP knockdown, suggesting TRAIP is required for stable kinetochore–microtubule (K-MT) attachment. TRAIP knockdown decreased the accumulation of Mad2 on centromeres, potentially explaining why oocyte maturation was not affected following formation of DNA lesions. Securin, a protein which was prevent from precocious degradation by Mad2, was down-regulated after TRAIP knockdown. Inhibition of TRAIP by microinjection of antibody into pro-metaphase I (pro-MI) stage oocytes resulted in precocious first polar body (PB1) extrusion, and live-cell imaging clearly revealed misaligned chromosomes after TRAIP knockdown. Taken together, these data indicate that TRAIP plays important roles in oocyte meiosis regulation.

Oocytes harboring integral genetic material are essential for successful human reproduction, however, oocyte meiosis is an error-prone process^1^. Inaccurate chromosome segregation causes aneuploidy, which can result in infertility, abortion, or birth defects^2^,^3^. Faithful chromosome segregation is guaranteed by the spindle assembly checkpoint (SAC) system, whose components include proteins of the Bub and Mad families^4^,^5^. When kinetochores and microtubules are not correctly attached, resulting in insufficient spindle tension, the SAC delays cell cycle progression until the errors are corrected^6^. During prometaphase, various checkpoint proteins accumulate at unattached kinetochores, generating a diffusible signal that induces formation of the mitotic checkpoint complex (MCC), the predominant inhibitor of the anaphase-promoting complex/cyclosome (APC/C)^7^,^8^. Once all chromosomes are attached, the SAC is inactivated and anaphase can proceed.

Exogenous and endogenous damage to maternal DNA can have severe consequences^9^,^10^,^11^. Consequently, oocytes have elaborate mechanisms for avoiding such defects^12^,^13^. Several lines of evidence have revealed the existence of cross-talk between the DNA damage response (DDR) and the SAC. In budding yeast, Mad2 is indispensable for prolonged G2/M arrest after a single DNA double-strand break (DSB)^14^. As a central regulator of DNA damage, ataxia-telangiectasia mutated (ATM) is not only important for the response to DSBs, but also regulates SAC activation, and DNA damage activates the SAC in an ATM-dependent manner^15^,^16^.

The tumor necrosis factor (TNF) receptor-associated factor (TRAF)-interacting protein (TRAIP) is a RING-type E3 ubiquitin ligase involved in tumor necrosis factor-α (TNF-α)-mediated NF-κB activation^17^,^18^. In both human and mouse, the protein is composed of 469 amino acids and encoded by 15 exons. Of 11 predicted spliced transcripts revealed by annotation analysis, only the longest transcript encodes TRAIP protein^19^. TRAIP is expressed in normal tissues, and at higher levels in highly invasive breast cancer cells^18^,^20^. Real Interesting New Gene (RING), coiled-coil (CC), and leucine-zipper (LZ) domains are present in TRAIP, and expression of a TRAIP mutant lacking the CC domain increases mitotic index and accelerates mitotic progression^21^. In keratinocytes, knockdown of TRAIP leads to a reduction in cell proliferation and arrest in G1/S phase^22^. In both mice and *Drosophila*, TRAIP knockout is embryonic lethal due to aberrant mitosis^23^,^24^. In human, TRAIP is identified as a primordial dwarfism associated gene and is involved in DDR during genome replication^25^.

Recent studies showed that TRAIP is important for both DNA repair and SAC regulation. In response to DNA damage, TRAIP is necessary for recruitment of RAP80 to damage sites, and its knockdown leads to defects in the classical DDR^26^. TRAIP is also a PCNA-binding ubiquitin ligase with an important role in protecting genome integrity after replication stress, and its function loss results in chromosome instability and reduced survival under this type of stress^27^. In HeLa cells, TRAIP localizes at the periphery of chromosomes and is necessary for regulation of SAC in mitosis. TRAIP knockdown by siRNA decreases the time from nuclear envelope breakdown (NEB) to anaphase onset and increases the rate of chromosome alignment defects^28^.

Because previous findings about TRAIP were obtained in mitotic cells, the function of this protein in mammalian oocyte meiosis remains enigmatic. In this study, we found that TRAIP was stably expressed and localized to the centromeres during mouse oocyte meiosis. TRAIP silencing by microinjection of specific siRNAs led to formation of DNA lesions, misaligned chromosomes, aneuploidy, and improper K-MT attachment. In addition, Mad2 and securin accumulation were impaired after TRAIP knockdown, and TRAIP inhibition after GVBD resulted in precocious first polar body extrusion. Together, these findings reveal the importance of TRAIP during mouse oocyte meiosis.

## Materials and Methods

### Antibodies

The goat polyclonal anti-TRAIP (sc-99695), anti-Mad2 (sc-6329) and mouse monoclonal anti-securin (sc-398471) antibodies were purchased from Santa Cruz Biotechnology, rabbit monoclonal anti-β-actin (4970) was brought from Cell Signal Technology, mouse monoclonal anti-BubR1 antibody (ab54894) was from Abcam, mouse monoclonal anti-α-tubulin-FITC antibody (F2168) was obtained from Sigma.

CREST, human anti-centromere antibody (HCT-0100) was brought from Immunovision. Anti-γH2AX antibody (05-636) was from Millipore.

FITC or TRITC conjugated goat anti-mouse, rabbit anti-goat antibodes and goat anti-human antibodies were bought from Zhongshan Golden Bridge Biotechnology (ZF-0314, ZF-0317, ZF-0313, ZF-0308). All other reagents were bought from Sigma Aldrich except when noted otherwise.

### Mouse oocyte culture, microinjection and live-imaging

Animal care and handling were conducted according with Animal Research Committee policies of the Peking University, and all the relevant protocols were approved by the Institutional Animal Care and Use Committee of Peking University Third Hospital. Female ICR mice 6-8 weeks old were sacrificed by cervical dislocation and the ovaries were isolated, the ovaries were then cut by blade to release immature oocytes, only the oocytes with an intact germinal vesicle were collected for further studies. For *in vitro* culture, oocytes were cultured in M2 medium under liquid paraffin oil at 37 °C in a 5% CO_2_ incubator.

Microinjection was performed by using Narishige micromanipulation system that was attached to an invert microscope and completed within 30 minutes. For TRAIP knockdown, small interfering RNAs against TRAIP (Genepharma) were microinjected into the cytoplasm of GV stage mouse oocyte, the siRNAs were diluted to 50 μM, the same amount of negative control siRNA was used as control. After microinjection, the oocyte were arrested at GV stage in M2 medium contain 2.5 μM milrinone for 24 hours for TRAIP knockdown.

For oocytes live-imaging, DNA was stained with Hoechst 33342 (10 nM). Image of live oocytes were acquired with a 20x objective on a spinning disk confocal microscope (Perkin Elmer), exposure time were set ranging from 300–800 ms depended on the fluorescence level of Hoechst 33342 and imaged at 5 minutes intervals for 10 hours.

### Western blot analysis

Samples contain at least 150 oocytes at appropriate stage were collected in 2X SDS loading buffer and boiled for 5 minutes. After separation by SDS-PAGE, the proteins were transferred to polyvinylidene fluoride (PVDF) membranes. After transfer, the membranes were washed briefly in TBST and then blocked in TBST containing 5% skim milk at room temperature for 1 hour, followed by incubation overnight at 4 °C with goat polyclonal TRAIP antibody or rabbit monoclonal anti-β-actin at a 1:200 dilution. After three washes in TBST buffer for 10 minutes each, the membranes were incubated at 37 °C for 1.5 hours with 1:1000 horseradish peroxidase (HRP)-conjugated rabbit anti-goat or mouse anti-rabbit antibody. Finally, the membranes were washed in TBST and processed using the enhanced chemiLuminescence detection system (Bio-Rad, CA).

### Immunofluorescence staining and chromosome spreads

Oocytes were fixed with 4% paraformaldehyde in PBS (PH 7.4) for 30 minutes at room temperature. After being permeabilized with 0.5% Triton X-100 at room temperature for 30 minutes, oocytes were blocked in PBS contain 1% BSA for 1 h and then incubated with goat polyclonal anti-Mad2, mouse monoclonal anti-securin or mouse monoclonal anti-BubR1 antibody (1:50) overnight at 4 °C, all diluted in washing buffer (PBS containing 0.1% Tween-20 and 0.01% Triton X-100). After incubation, oocytes were washed for three times in washing buffer for 5 minutes each, the oocytes were further incubated in second antibody (1:100) according to the host of the primary antibody at 37 °C for 1.5 hours, oocytes were finally stained with Hoechst 33342 for 10 minutes to visualize the nucleus.

Chromosome spreads staining were performed as described previously. Briefly, after the zona pellucida was removed, the oocytes were fixed in a solution of 1% paraformaldehyde in distilled water (pH 9.2) containing 0.15% Triton X-100 and 3 mM dithiothreitol. The slides were dried slowly at room temperature for several hours, and then blocked with 1% bovine serum albumin (BSA) prepared in PBS for 1 hour at room temperature. Oocytes were then incubated with anti-TRAIP (1:50) overnight at 4 °C. After brief washes with washing buffer, the slides were then incubated with the according secondary antibodies for 2 hours at room temperature. For double staining of TRAIP and CREST, the slides were first stained with CREST antibody (1:50) overnight at 4 °C after blocked, the slides were then incubated with goat anti-human antibody (1:100) for 2 hours, then washed three times and blocked again for 1 hour at room temperature and then stained for TRAIP as previously. DNA on the slides were stained with Hoechst 33342 for 10 minutes. All photos were taken with confocal laser- scanning microscope (Zeiss LSM 710, Germany).

ImageJ software (National Institutes of Health, USA) was used to evaluate the immunostaining signal of securin on spindles. The red signal were chosen and assessed by ImageJ software for comparison. All photos were taken under the same conditions.

### Cold treatment assay for kinetochore microtubule attachment

This assay was conducted according to a previously published method. Briefly, after TRAIP knockdown by siRNA microinjection, the oocytes were arrested at GV stage for 24 hours, after washed and cultured in M2 medium for 8 hours, oocytes were transferred to pre-cooled 4 °C M2 medium for 10 minutes and then collected for immunofluorescence staining for kinetochore and α-tubulin.

### Statistical analysis

All experiments were performed independently at least for three times, all percentage data were presented as mean ± SEM. Data were analyzed by Chi-square test with SPSS software (SPSS Inc., Chicago, IL). P < 0.05 was considered statistically significant.

## Results

### Expression and localization of TRAIP during mouse oocyte maturation

**Figure 1:**
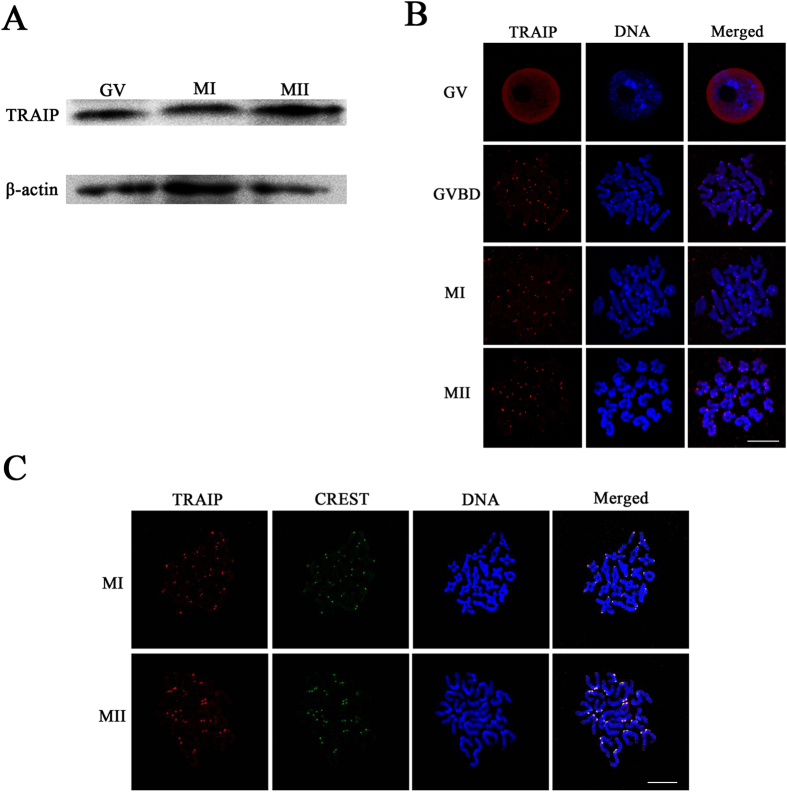
The expression and subcellular localization of TRAIP during mouse oocyte meiosis. (**A**) The expression of TRAIP during mouse oocyte meiotic maturation was detected by West blotting. Oocytes were collected after 0, 8 and 14 hours after cultured, corresponding to GV, MI and MII stage, respectively. The molecular weight of TRAIP and β-actin were 53 KD and 43 KD, respectively. (**B**) Subcellular localization of TRAIP was revealed by immunoflourescence and confocal microcopy. GV, GVBD, MI and MII stage oocytes were stained. TRAIP was stained in red and the Hoechst 33342 was used to visualize the DNA (blue), bar = 10 μm. (**C**) Co-localization of CREST and TRAIP at the centromere region at the MI and MII stage oocytes. TRAIP was stain in red and CREST was stained in green, bar = 10 μm.

Expression of TRAIP during mouse oocyte meiotic maturation was detected by western blot. For this purpose, a total of 150 oocytes were collected after culture *in vitro* for 0, 8 or 14 hours, corresponding to the germinal vesicle (GV), the first metaphase (MI), and the second metaphase (MII) stages, respectively. The level of TRAIP was stable throughout oocyte maturation (Fig. 1A).

The localization of TRAIP was checked by immunofluorescence analysis after chromosome spreading. At the GV stage, TRAIP concentrated in the nucleus, whereas after GVBD, staining appeared on the centromeres. At MI and MII, TRAIP was still localized on the centromeres (Fig. 1B). To further confirm the centromeric localization of TRAIP, we double stained for CREST (anti-centromere antibody) and TRAIP, and found that the signals overlapped (Fig. 1C). Based on these observations, we hypothesized that TRAIP has a function in chromosome alignment and kinetochore–microtubule attachment.

### TRAIP knockdown does not affect meiosis progression, but does cause DNA lesions and chromosome misalignment

**Figure 2:**
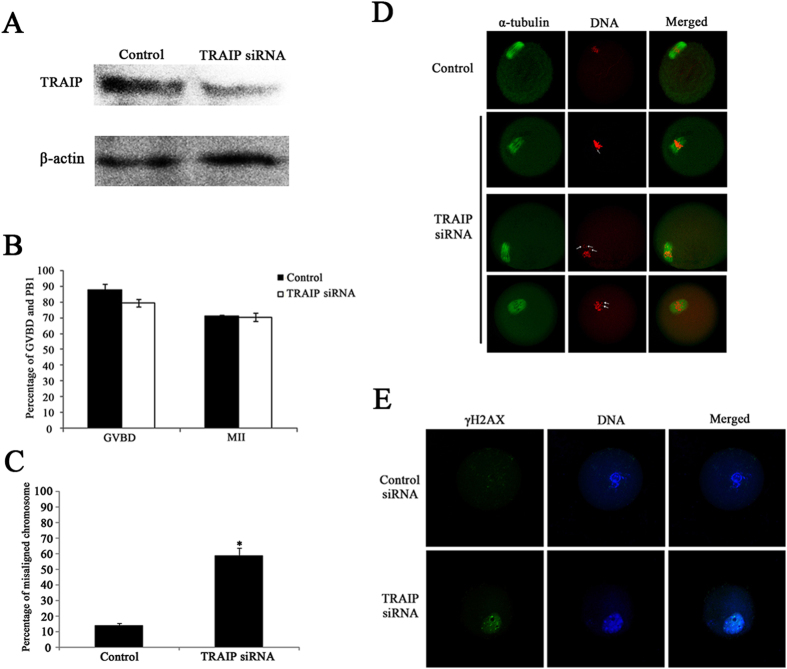
Knockdown of TRAIP caused misaligned chromosomes and DNA damage. Oocytes injected with control or TRAIP siRNAs were arrested at GV stage in M2 medium containing 2.5 μM milrinone for 24 hours, and then washed and cultured in M2 medium for further studies. (**A**) West blotting indicated TRAIP was notable knockdown after specific siRNAs microinjection. A total of 150 oocytes were loaded in each lane. (**B**) The GVBD and PB1 extrusion ratio in control group (n = 269) and TRAIP knockdown (n = 160) group were calculated following 3 and 14 hours cultured. The data were presented as mean ± SEM. (**C,D**) The morphology of spindle organization and chromosome alignment (**D**), and the percentage of misaligned chromosomes in control (n = 145) and TRAIP knockdown group (n = 129) after 14 hours cultured (**C**). The arrowheads indicated misaligned chromosomes in TRAIP knockdown oocytes while the chromosomes in control group aligned well. The data were presented as mean ± SEM. A asterisk indicated p < 0.05, bar = 50 μm. (**E**) γH2AX was occurred in the nucleus of GV stage oocytes after TRAIP knockdown while was invisible in the control group oocytes, bar = 50 μm.

To explore the role of TRAIP in mouse oocyte meiotic maturation, we knocked down its expression by microinjecting TRAIP-specific siRNAs into GV stage oocytes. After microinjection, the oocytes were arrested at GV stage for 24 hours in M2 medium containing 2.5 μM milrinone. Western blots confirmed that the expression of TRAIP was significantly reduced by specific siRNAs (Fig. 2A). To determine the effect of TRAIP knockdown on oocyte meiotic progression, we monitored GVBD and the first polar body (PB1) extrusion after 3 and 14 hours of *in vitro* culture, respectively. Curiously, the proportion of oocytes undergoing GVBD (79.4 ± 2.3% vs 87.8 ± 3.4%, p > 0.05) and PB1 extrusion (70.4 ± 2.5% vs 71.0 ± 0.7%, p > 0.05) did not differ significantly between TRAIP-depleted and control siRNA-treated oocytes (Fig. 2B).

Given that TRAIP is associated with DNA damage and SAC regulation, we next analyzed DNA lesions, spindle organization, and chromosome alignment in TRAIP-depleted oocytes. We performed immunofluorescence staining to visualize phosphorylated H2AX (γH2AX), a widely used marker for DNA damage, in control and TRAIP knockdown oocytes. γH2AX was almost completely absent in control oocytes, but abundant in the nucleus of TRAIP knockdown oocytes (Fig. 2E). Thus, TRAIP knockdown in oocytes caused formation of DNA lesions. In addition, TRAIP knockdown interfered with chromosome alignment, but had no effect on spindle organization (Fig. 2D). Specifically, the proportion of oocytes exhibiting abnormal chromosome alignment was significantly higher in TRAIP siRNA oocytes than in control group (Fig. 2C, 58.7 ± 5.0% vs 13.6 ± 1.8%, p < 0.05). Together, these findings demonstrated that TRAIP knockdown results in formation of DNA lesions and chromosome misalignment.

### TRAIP knockdown increases the rate of aneuploidy and disrupts K-MT attachment

**Figure 3:**
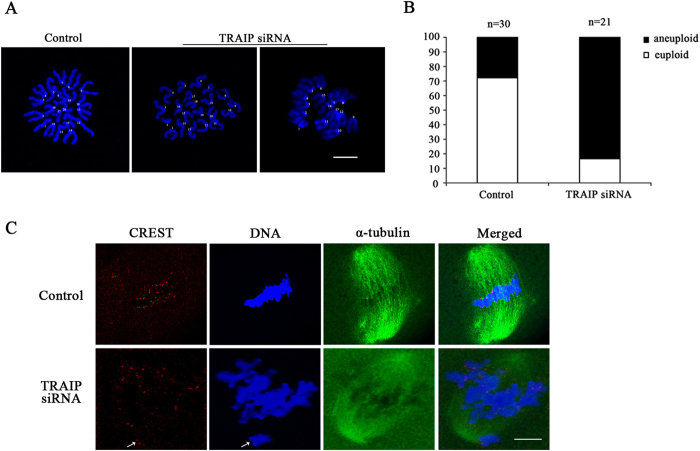
The incidence of aneuploidy was increased and K-MT attachment was disorder after TRAIP knockdown. (**A**) Chromosome spreading was employed to analyze chromosome numbers after TRAIP knockdown. There were 20 pairs of sister chromosomes in the control group, but the count was more or less than 20 pairs after TRAIP knockdown (as indicated by 21 and 15 pairs), bar = 10 μm. (**B**) Quantification of aneuploidy after TRAIP knockdown, the ratio of aneuploidy was 14/21 and 4/30 in TRAIP knockdown and the control group, respectively. (**C**) The control and TRAIP knockdown oocytes were cold-treated after 8 hours cultured, CREST (red) and α-tubulin (green) were stained to reveal K-MT attachment, Hoechst 33342 was used to visualize the DNA. Kinetochores were attached to microtubules and well aligned in the control group, but it was disconnect from the microtubules after TRAIP knockdown when processing with cold treatment. The arrowhead indicated disconnected kinetochore and chromosome, bar = 10 μm.

It has been reported that misaligned chromosome frequently caused aneuploidy in mouse oocytes. Due to the chromosomes were misaligned after TRAIP knockdown, we investigated whether TRAIP knockdown was also related to aneuploidy formation. To this end, we employed chromosome spreading to determine exact chromosome number in MII stage oocytes following TRAIP knockdown. In the control group, most oocytes contained 20 pairs of sister chromosomes, whereas the proportion of aneuploid was significantly elevated in TRAIP-depleted oocytes (Fig. 3A,B. 13.3% vs 66.7%).

Misaligned chromosomes and aneuploidy can both be caused by improper K-MT attachment. Appropriate K-MT attachment is stable after cold treatment, whereas inappropriately attached K-MT complexes are disassembled at low temperatures^29^. Therefore, we subjected oocytes to cold treatment and checked K-MT attachment after TRAIP knockdown. In control groups, microtubules were attached to kinetochores (as revealed by immunofluorescence staining by CREST) and the chromosomes were well aligned, whereas in TRAIP-depleted groups, some kinetochores disconnected and disassociated from the spindles (Fig. 3C). These results indicated that TRAIP is required for proper K-MT attachment, and thus for proper chromosome alignment and accurate chromosome segregation.

### Localization of Mad2, a component of the SAC, is disrupted by TRAIP knockdown

**Figure 4:**
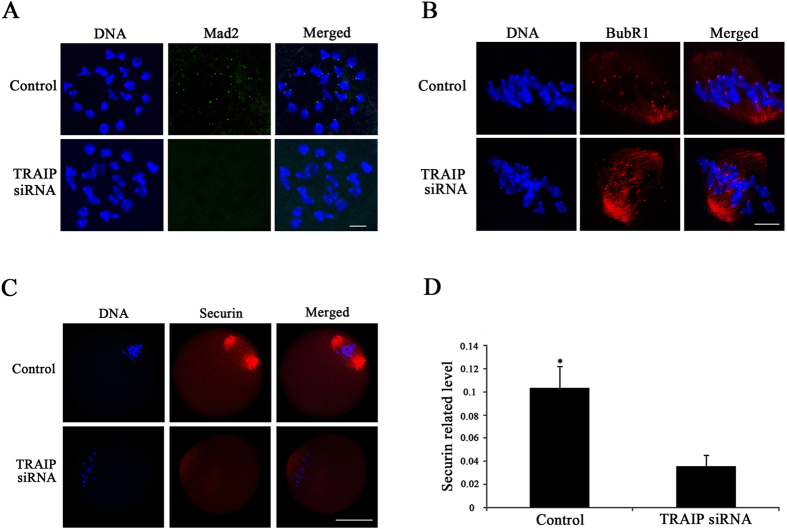
TRAIP knockdown selectively reduced the amount of Mad2 on the kinetochores. (**A**) The control and TRAIP knockdown oocytes were stained with Mad2 (green) antibody after 6 hours cultured. Mad2 was recruited on the chromosomes in the control group while was invisible after TRAIP knockdown, bar = 10 μm. (**B**) The localization of BubR1(red) was not affected after TRAIP knockdown when compared with control group, bar = 10 μm. (**C**) Securin localization was investigated in control and TRAIP knockdown oocytes after 8 hours cultured. In the control group, secuin located on the spindles (red), but was faint stained after TRAIP knockdown, bar = 50 μm. (**D**) Quantification of securin signals in the control and TRAIP knockdown oocytes. The data were presented as mean ± SEM. A asterisk indicated p < 0.05.

The results presented above indicated that misaligned chromosomes could evade the checkpoint in TRAIP-depleted oocytes, preventing a delay in meiotic progression. To explain this observation, we hypothesized that SAC function was impaired by TRAIP knockdown. To determine which component of the SAC was affected by TRAIP knockdown, we investigated the localization of BubR1 and Mad2. TRAIP knockdown drastically decreased the accumulation of Mad2 on chromosomes, but had no effect on BubR1 localization (Fig. 4A,B). These results implied that TRAIP selectively regulates accumulation of SAC components, consistent with a previous study performed in mitotic cells^28^.

We next investigated the localization of securin (a target protein of Mad2) after TRAIP knockdown. In control groups, securin was accumulated on the spindles, but this accumulation was significantly diminished by TRAIP knockdown (Fig. 4C,D). Taken together, these results demonstrated that TRAIP is required for recruitment of Mad2 and its downstream target during oocyte meiosis.

### TRAIP inhibition at pro-MI stage leads to acceleration of PB1 extrusion

**Figure 5:**
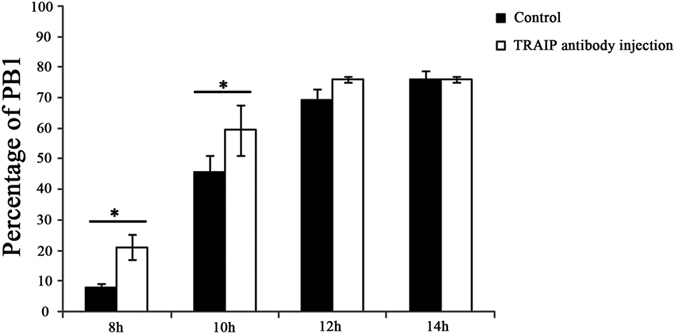
TRAIP inhibition at pro-MI stage oocytes caused precocious PB1 extrusion. The oocytes were cultured for 6 hours corresponding to pro-MI stage and then microinjected with normal IgG or TRAIP antibody. The PB1 extrusion was checked at 2 hours interval until 14 hours (before and after antibody injection). The results suggested that PB1 extrusion was significantly increased at 8 and 10 hours while not 12 and 14 hours after TRAIP inhibition when compared with the control group(n = 88 and 224 for TRAIP inhibition and control group, respectively). The data were presented as mean ± SEM. A asterisk indicated p < 0.05.

In mouse oocytes, DNA damage induces MI arrest but does not block GVBD^30^,^31^. As we showed above, however, PB1 was not affected by the DNA lesions induced by TRAIP knockdown. We hypothesized that first meiosis might be accelerated when TRAIP was knockdown. To this end, we inhibited TRAIP function in pro-MI stage oocytes by microinjection of anti-TRAIP antibody, cultured the microinjected oocytes for a total of 14 hours, and quantitated PB1 extrusion at 8, 10, 12, and 14 hours after injection (Fig. 5). PB1 extrusion was significantly elevated relative to the control at 8 hours (21.2 ± 4.0% vs 7.9 ± 1.4%, p < 0.05) and 10 hours (59.5 ± 8.3% vs 45.9 ± 5.4%, p < 0.05) after TRAIP inhibition, but no differences were observed at 12 hours (76.0 ± 1.1% vs 69.1 ± 3.6%, p > 0.05) and 14 hours (76.0 ± 1.1% vs 76.0 ± 2.7%, p > 0.05). Thus, TRAIP inhibition at pro-MI stage during oocyte meiosis accelerated first meiosis.

### Live-cell imaging confirms the role of TRAIP in chromosome alignment

**Figure 6:**
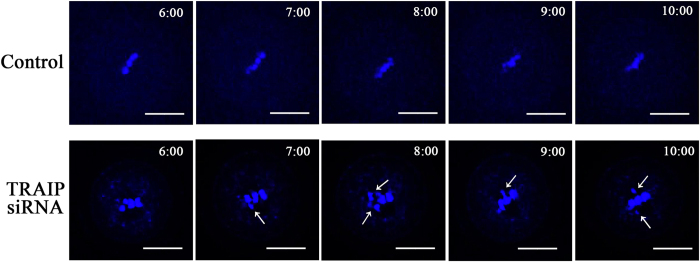
TRAIP knockdown caused chromosome misalignment revealed by time-lapse live-cell imaging. The control and TRAIP knockdown oocytes were cultured and the chromosomes alignment was monitored. Images indicated chromosomes aligned well along with meiosis progression in the control group, chromosome were misaligned after TRAIP knockdown, time points indicated the cultured duration, the arrowhead indicated disassociation chromosome, bar = 50 μm.

To further elucidate the function of TRAIP in chromosome alignment during mouse oocyte meiosis, we employed time-lapse live-cell imaging. In control oocytes, chromosomes were dynamically changing and could be well aligned after *in vitro* cultured (Fig. 6, Supplemental Video 1). By contrast, in TRAIP-depleted ooccytes, a subset of chromosomes dissociated from the equatorial plate, potentially resulting in formation of aneuploidy (Fig. 6, Supplemental Video 2).

## Discussion

Previous work identified TRAIP as a human disease-associated target protein and is involved in DDR and SAC regulation^25^,^26^,^27^,^28^. However, the specific role of TRAIP in mammalian oocyte meiosis had not been explored in detail. In this study, we characterized the expression, localization, and function of TRAIP in mouse oocyte meiosis. In oocytes, TRAIP localized to centromeric regions, and its knockdown resulted in formation of DNA lesions, chromosome misalignment, and aneuploidy. Moreover, knockdown of TRAIP decreased recruitment of Mad2 to the centromere, thereby reducing the level of securin in the spindle region. Together, these results illustrated the crucial role of TRAIP in mouse oocyte meiosis.

In HeLa cells, the localization of TRAIP was discrepantly reported. Specifically, mCherry-TRAIP in HeLa cells exhibits a punctate distribution in the nucleus distinct from that of CREST^24^. Immunofluorescence staining indicated that TRAIP diffused throughout the cell at prophase and become around prometaphase chromosomes while colocalization with anaphase chromosomes^28^. In this study, we were able to determine TRAIP localization in oocytes by using the chromosome spreading method. The results revealed that TRAIP was concentrated in the nucleus, located mainly in centromeric regions during oocyte meiosis. The discrepancies in these results might be due to differences in cell types or experimental methods. Nonetheless, our results revealed the specific localization of TRAIP and suggested that this protein has an important function in mouse oocyte meiosis.

TRAIP is associated with regulation of the DDR and SAC in mitosis^26^,^27^,^28^. Therefore, we investigated whether TRAIP is also involved in these processes in mouse oocytes. To this end, we monitored γH2AX status and several morphological parameters in TRAIP-depleted mouse oocytes. The results revealed that γH2AX appeared after TRAIP knockdown in mouse oocytes. In mitosis, TRAIP is required for RAP80 recruitment to DNA damage sites, and as a PCNA-binding protein; it is indispensable for genome integrity after replication stress^26^,^27^. We hypothesized that the functional proteins involved in the DDR were impaired after TRAIP knockdown in mouse oocytes, resulting in deficient DNA repair; however, this phenomenon has yet to be explored in detail in oocytes. Our observations also revealed that the rates of chromosome misalignment and aneuploidy were elevated, whereas spindles were unaffected, following TRAIP knockdown in mouse oocytes. Similarly in TRAIP-knockdown HeLa cells, chromosomes are misaligned, although the cells still have bipolar spindles^28^. Because misaligned chromosomes and aneuploidy are frequently caused by improper K-MT attachment^32^, and unstable K-MT attachments disassemble at low temperature^29^, we then subjected TRAIP-depleted oocytes to cold treatment and checked the K-MT attachment. The results revealed that the K-MT attachment was defective after TRAIP knockdown in mouse oocytes, and the underlying mechanisms might be related to DNA damage altered kinetochore function^14^.

Although DNA damage does not affect GVBD, it blocks anaphase-promoting complex (APC) activity and consequently suppresses oocyte maturation^31^. In both mitosis and meiosis, APC activity is inhibited by the SAC^33^. To further explore the function of TRAIP during oocyte meiotic progression, we monitored GVBD and PB1 extrusion after TRAIP knockdown. The results revealed that TRAIP knockdown inhibited neither resumption of oocyte meiosis nor PB1 extrusion. The observation of DNA damage did not block oocyte meiosis resumption was partially consistent with the earlier studies, but the PB1 extrusion was not inhibited after TRAIP knockdown puzzled us. Since the morphology of TRAIP-knockdown in oocytes was similar to that of SAC impaired, we assumed that PB1 extrusion was not suppressed due to impairment in the SAC, and thus caused the first meiosis was accelerated by TRAIP knockdown.

The major components of the SAC are members of the Bub and Mad protein families. Knockdown of SAC components results in acceleration of oocyte maturation and several types of meiotic defects^34^,^35^,^36^. Therefore, we investigated the effect of TRAIP knockdown on two well-characterized SAC components, Mad2 and BubR1. Localization of Mad2, but not BubR1, was altered by TRAIP knockdown. These observations are consistent with our hypothesis that the SAC in oocytes is impaired by TRAIP knockdown. Moreover, these observations are also consistent with findings made in HeLa cells, TRAIP knockdown accelerated the cell cycle from NEB to onset of anaphase onset, to our surprise, this study also concluded that the accumulation of Mad2, but not other SAC components, were reduced after TRAIP knockdown^28^. The mechanisms by which TRAIP selectively regulates Mad2 accumulation remain unknown and should be addressed in future studies. Collectively, our findings indicate that TRAIP knockdown in oocytes selectively regulates Mad2 accumulation and accelerates first meiosis, and consequently has no effect on PB1 extrusion after DNA damage occurs.

The results described above, particularly in relation to formation of DNA lesions and decreased Mad2 localization after TRAIP knockdown, indicate that TRAIP plays a complex role in oocyte maturation. In mouse oocytes, the level of securin continues to increase in response to DNA damage, indicating insufficient APC activity^31^. Meanwhile, Mad2 inhibits APC activity and prevents premature proteolysis of securin by the APC^36^,^37^,^38^. These observations imply that securin is an appropriate substrate with which to highlight the intricate role of TRAIP during mouse oocyte maturation. In this study, the localization of securin was investigated after TRAIP knockdown and the results indicated securin was faint stained after TRAIP knockdown in mouse oocytes. The down-regulation of securin upon TRAIP knockdown implies that TRAIP is likely to regulate the SAC and inhibit APC activity during oocyte maturation.

Previous studies showed that oocyte MI arrest is statistical reduced when DNA damage is induced after GVBD than when it is induced at the GV stage^31^. This led us to investigate the effect of TRAIP inhibition after GVBD on oocyte maturation, and in particular to ask whether TRAIP disruption after GVBD could cause precocious PB1 extrusion. To this end, TRAIP antibody was microinjected into pro-MI stage oocytes and the PB1 extrusion was monitored. In TRAIP-inhibited oocytes, PB1 extrusion was increased at 5 and 7 hours after GVBD. We attributed the temporal role of TRAIP on oocyte maturation was that the DNA damage occurred after GVBD was not adequate to induce oocyte MI arrested.

In summary, TRAIP plays crucial roles in DNA damage, chromosome alignment, and SAC regulation in mouse oocytes. Because TRAIP is mutated in primordial dwarfism patients, these findings might improve our understanding of this catastrophic human disease.

## Additional Information

**How to cite this article**: Yuan, Y.-F. *et al*. TRAIP is involved in chromosome alignment and SAC regulation in mouse oocyte meiosis. *Sci. Rep.* **6**, 29735; doi: 10.1038/srep29735 (2016).

## Supplementary Material

Supplementary Information

Supplementary Video S1

Supplementary Video S2
