# Supplementary material for: TRAIP is involved in chromosome alignment and SAC regulation in mouse oocyte meiosis
Source: Sci Rep. 2016 Jul 11;6:29735. doi: 10.1038/srep29735 (PMC4942609; doi:10.1038/srep29735)
Supplement: Supplementary Information [file srep29735-s1.doc]

**Supplementary information**

**TRAIP is involved in chromosome alignment and SAC regulation in mouse oocyte meiosis**

Yi-Feng Yuan^1,2^, Yi-Xin Ren^2^, Peng Yuan^2^, Li-Ying Yan^1,2^ and Jie Qiao^1,2,*^

^1^ Center for Reproductive Medicine, Department of Obstetrics and Gynecology, Peking University Third Hospital, No.49 North HuaYuan Road, HaiDian District, Beijing 100191, China

^2^ Key Laboratory of Assisted Reproduction, Ministry of Education, Beijing 100191, China

^*^Author for correspondence

E-mail: jie.qiao: [jie.qiao@263.net](mailto:jie.qiao@263.net)

**Video 1. The dynamical of chromosomes alignment in control oocyte.** Chromosomes were dynamically changing and could be well aligned in the control group.

**Video 2. The dynamical of chromosomes alignment in TRAIP knockdown oocyte.** A subset of chromosomes dissociated from the equatorial plate after TRAIP knockdown.
